# Chitosan-Based Nano-Smart Drug Delivery System in Breast Cancer Therapy

**DOI:** 10.3390/pharmaceutics15030879

**Published:** 2023-03-08

**Authors:** Yedi Herdiana, Nasrul Wathoni, Dolih Gozali, Shaharum Shamsuddin, Muchtaridi Muchtaridi

**Affiliations:** 1 Department of Pharmaceutics and Pharmaceutical Technology, Faculty of Pharmacy, Universitas Padjadjaran, Sumedang 45363, Indonesia; 2 School of Health Sciences, Universiti Sains Malaysia, Kubang Kerian 16150, Malaysia; 3 Nanobiotech Research Initiative, Institute for Research in Molecular Medicine (INFORMM), USM, Penang 11800, Malaysia; 4 USM-RIKEN Interdisciplinary Collaboration on Advanced Sciences (URICAS), USM, Penang 11800, Malaysia; 5 Department of Pharmaceutical Analysis and Medicinal Chemistry, Faculty of Pharmacy, Universitas Padjadjaran, Sumedang 45363, Indonesia

**Keywords:** nanocarrier, multimodal delivery, stimulus response, targeting

## Abstract

Despite recent advances, cancer remains the primary killer on a global scale. Numerous forms of research have been conducted to discover novel and efficient anticancer medications. The complexity of breast cancer is a major challenge which is coupled with patient-to-patient variations and heterogeneity between cells within the tumor. Revolutionary drug delivery is expected to provide a solution to that challenge. Chitosan nanoparticles (CSNPs) have prospects as a revolutionary delivery system capable of enhancing anticancer drug activity and reducing negative impacts on normal cells. The use of smart drug delivery systems (SDDs) as delivering materials to improve the bioactivity of NPs and to understand the intricacies of breast cancer has garnered significant interest. There are many reviews about CSNPs that present various points of view, but they have not yet described a series in cancer therapy from cell uptake to cell death. With this description, we will provide a more complete picture for designing preparations for SDDs. This review describes CSNPs as SDDSs, enhancing cancer therapy targeting and stimulus response using their anticancer mechanism. Multimodal chitosan SDDs as targeting and stimulus response medication delivery will improve therapeutic results.

## 1. Introduction

GLOCAN has reported 20 million additional cancer diagnoses and 10 million deaths in 2020. Breast cancer (BC) ranks first compared to other cancers [1]. Current therapies are still ineffective in eradicating the disease as a whole, and therefore require improvement using far more specific treatments [2]. Current conventional chemotherapy uses poorly water-soluble drugs with limited delivery to target tissues, develops resistant tumors, has high drug toxicity in normal cells, and causes severe side effects, rapid degradation, low specificity, and limited targeting [3,4,5,6,7,8,9]. Drug development and drug delivery systems are the main challenges in cancer therapy [8,10]. 

Encapsulation or trapping of drugs in nanocarriers can increase their solubility, facilitate their transportation in circulation to cancerous tissues, maintain anticancer potential, minimize drug toxicity by reducing inappropriate distribution, and increase drug accumulation at the targeted site, increase bioavailability, and extend a drug’s half-life [2,5,11,12]. The nanovehicles concepts that form the basis of the chemical approach to nanomaterials are small size, large surface area, high drug-loading capacity, easy surface functionalization and increased stability of nanoparticle formulations, making polymer-based NPs an efficient delivery platform [6,13]. Recent investigations have proven the efficacy of NT in cancer treatment by designing a wide variety of nanovehicles (NVs). There are many choices in the development of nanovehicles, such as liposomes, dendrimers, polymeric nanoparticles, carbon nanotubes, iron oxide nanoparticles, and gold nanoparticles. Advances in NT-based delivery systems have allowed the simultaneous delivery of different anticancer agents, including drug–drug or gene–drug combinations [14,15,16].

The hallmark properties of polymeric nanoparticles as cancer nanovehicles have been proven in various research including targeted drug delivery, controlled drug release, enhanced therapeutic efficacy and safety [17,18,19,20,21]. Efforts in the development of drug delivery systems for chemotherapy have been focused on delivering chemotherapy drugs directly to cancer cells while minimizing toxic effects on healthy tissue. This strategy has the potential to increase treatment efficacy and reduce side effects [22,23,24]. Polymeric NPs (PNPs) are submicron colloidal particles broadly used in drug delivery systems, owing to the relatively easy attachment of targeting ligands to the surface of NPs. Surface modification of these PNPs with targeting ligands enables recognition by specific receptors or ligand-binding sites that are overexpressed on cancer cells or at target sites, for the controlled release of loaded drugs [25,26,27,28]. The rational design of targeted drug delivery systems based on PNPs involves taking into consideration their composition, solubility, crystallinity, molecular weight, backbone stability, hydrophobicity and polydispersity. The polymeric shell of PNPs protects the drugs from enzymatic degradation. Furthermore, hydrophilic chains can be attached to the outer surface of the PNPs to avoid the process of phagocytosis and opsonization by circulating phagocytic cells. There are several advantages of using natural polymers as nanovehicles in cancer therapy: biocompatibility, biodegradability, targeting moieties, low immunogenicity, and cost-effectiveness [29,30,31]. Polymeric nanoparticles are a promising drug delivery system for chemotherapy; however, several challenges need to be addressed to optimize their effectiveness. These challenges include achieving optimal drug loading, achieving stable drug release, and preventing the development of drug resistance in cancer cells [22,24,32,33]. Notably, several types of polymers have been employed for this purpose, including polyethylene glycol (PEG), poly(lactic-co-glycolic acid) (PLGA), chitosan, poly(ethyleneimine) (PEI), poly(methacrylate) (PMA), and poly(N-isopropylacrylamide) (PNIPAM). 

Chitosan, a biodegradable and biocompatible polysaccharide derived from chitin, has been extensively studied as a promising nanovehicle for cancer therapy. Chitosan has shown potential in cancer therapy, especially as a dissolving agent, drug stabilizer, and controlled-release drug control, with a multifunctional platform for targeting, stimulus-responsive release, or use in image-guided medicine [34]. CSNPs will meet the design criteria for achieving cancer-targeting goals, which include (1) selective targeting of cancer cells, (2) efficient anticancer drug release at target sites, and (3) elimination of cytotoxicity to non-cancerous tissues [35]. Compared to other natural polymers such as alginate or gelatin, chitosan has a unique combination of properties that make it a promising option for cancer therapy. However, the optimal choice of polymer for a specific nanomedicine application will depend on the specific requirements and goals of the application, and will also consider factors such as biocompatibility, drug compatibility, and ease of production. 

There are many reviews about CSNPs that present various points of view, but they have not yet described a series in cancer therapy from cell uptake to cell death. We consider this knowledge very important in designing chitosan-based preparations. This review focuses on CSNP-based SDD approaches to enhance the effectiveness and efficiency of cancer therapy through stimulus-responsive particle engineering, passage targeting, and drug release mechanisms. Smart polymer rational design will improve drug therapy and reduce side effects, thereby providing more effective and cost-effective treatment and accelerating patient recovery.

## 2. General Overview of Breast Cancer

### 2.1. Features of Breast Cancer

BC is a global health challenge and tends to be more aggressive [5,36]. Despite improved preventative measures and treatment options, recurrence risk is 20–30%, with 5–7% of BC patients having metastatic illness later in life [37]. On the other hand, since cancer causes are complex, a single therapeutic approach is usually insufficient to suppress cancer cell proliferation, growth, and migration [38]. 

### 2.2. Breast Cancer Classification

BC is identified by the presence or absence of three main surface receptors. These include estrogen, progesterone, and hEGF-2 receptors (HER2). They identified four subgroups of BC: luminal A and luminal B, expressing ER, basal-like, and HER2-enriched, without ER or PR expression. Surrogate BC classification is common in practice. This classification includes five molecular and histological BC types. Tumor biology to molecular targeting changed with this inherent categorization. Surface receptors may also characterize BC. HER2-positive BC lacks ER or PR receptors. Hormone therapy will not work on HER2 cancer. ER, PR and HER2 are absent. It is not treatable with hormonal or HER2-targeted therapy. The prognosis for TNBC is dismal, owing to the lack of targeted therapy. TNBC is presently only chemo-treated. Localized TNBC had a 5-year survival rate of 91%, locally progressed TNBC had one of 66%, and metastatic TNBC one of 11% [20]. The most aggressive subtype of TNBC can metastasize to the lung and brain [39].

BC therapy has two alternatives. This therapy involves localized and systemic treatment. The therapy choices depend on the cancer’s stage and kind (molecular and histological categorization). If the tumor is contained in the breast tissue or lymph nodes, surgery or radiation can heal it. MBC is treated with chemotherapy, hormones, and targeted therapy.

### 2.3. Tumor Microenvironment 

**Figure 1:**
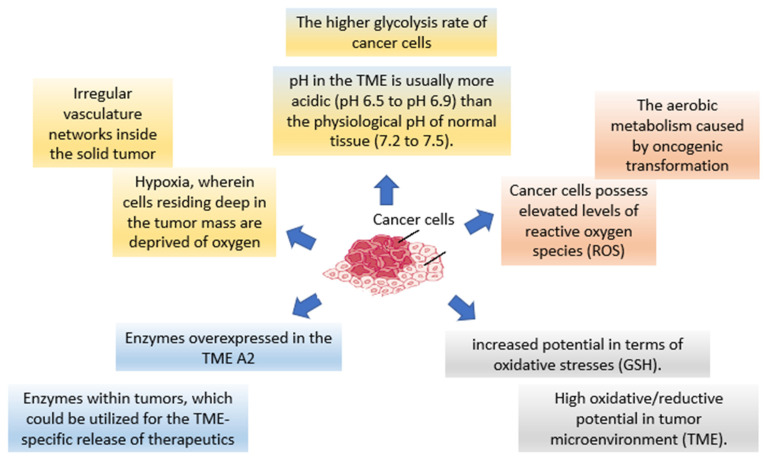
The opportunity for the development of TME-activatable NPs as “smart” technologies.

Tumor cells develop, by nature, in an uncontrolled manner. This results in forming a unique ‘tumor microenvironment’ which is conducive to tumor cell growth and development [22]. This includes alterations to the circulatory system, oxygenation, perfusion, pH, and metabolic rate. Fibroblasts, immune cells, the extracellular matrix, cytokines, and macrophages comprise the tumor microenvironment (TME). These cells alter the microenvironment and influence whether a tumor is pro- or anti-tumor. All of these components have the potential to interact with cancer cells, contributing to the process of carcinogenesis. The tumor microenvironment (TME), which consists of stromal cells and matrix components, is regarded to represent a significant impediment to the delivery of nanomedicines. TME contains unique enzymes (e.g., matrix metalloproteinases [MMPs]), higher amounts of glutathione (GSH), lower pH values, and hypoxic conditions and charge reversal (Figure 1).

### 2.4. Folate Receptors

The folic acid receptor alpha’s surface overexpression has been exploited to target cancer cells (FR). Due to quick proliferation, morphological changes, and the tumor microenvironment, cancer cells require more FA than normal cells and overexpress FR. This receptor is internalized after binding its ligand, and is recycled to the surface, allowing for ongoing drug target enrichment in malignant cell membranes. Many delivery strategies utilize the difference in glutathione content between the intracellular and extracellular environments to selectively release drugs [40]. 

FR-based delivery vehicles are internalized by target cells through receptor-mediated endocytosis. As a result, drug-loaded nanocarriers may be tailored to target particular cancer cell receptors, employing targeting ligands such as folic acid (FA) [41,42,43]. FA is a well-known vitamin for cell proliferation and biomarker targeting. FA-functionalized formulations may boost the therapeutic advantages of an anticancer medication (curcumin). Folate-conjugated nanomaterials have gained popularity in cancer biology, particularly in diagnostics and targeted medicine administration [44].

## 3. Chitosan-Based Nanosystem of Smart Drug Delivery System

**Figure 2:**
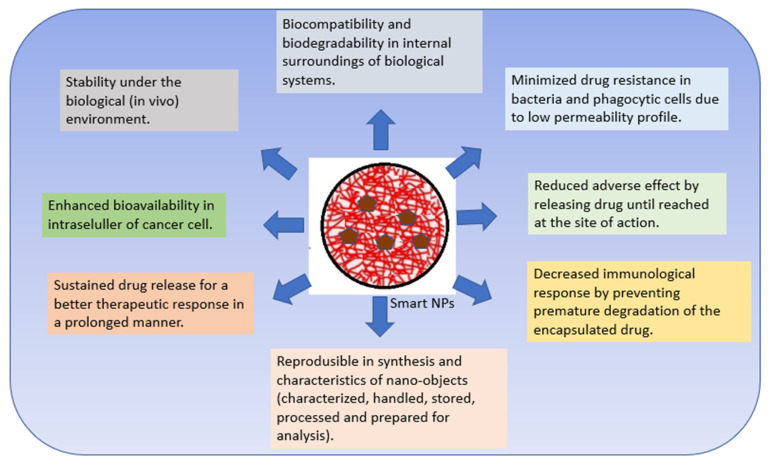
Characteristics of smart nanosystems-based carriers.

Polymer nanomaterials have potential in medical treatments and therapies such as imaging, faster diagnosis, and drug distribution, and offer many attractive advantages over conventional drugs, including stability, better bioavailability, minimized toxic side effects, and enhanced drug delivery [22,45,46]. Polymeric NPs are capable of combining other functions such as controlled release, imaging agency, targeted delivery, and the loading of more than one drug for combination therapy [47,48]. Numerous studies completed over the past two decades have emphasized the importance of smart polymers in biomedicine. Smart polymers are polymers that respond to stimuli and/or environmental conditions [49,50]. CSNPs are polymers that have attracted great attention as nanocarriers with multiple multi-functionalities in forming smart DDS [6]. Bioactive compounds can interact chemically or physically with other molecules. The intelligent response of a polymer is governed by its initial state [49,51]. Smart devices boost therapeutic effects and effectiveness in cellular and animal models compared to free drug and passive nanomaterial systems. As these technologies advance and more triggering and targeting approaches emerge, smart polymer nanoparticles will enhance cancer therapy [47,48]. Smart nanosystems-based carriers possess the following characteristics (Figure 2) [50,52,53,54,55,56].

### 3.1. Synthesis, Functionalization and Characterization of Chitosan Nanoparticles 

Chitosan is a deacetylated process derived from chitin, and consists of D-glucosamine and N-acetyl-D-glucosamine units linked to β-(1→4) [57,58]. Its cationic property, which enhances adhesion through electrostatic interactions to negatively charged mucosal surfaces, is one of the most significant characteristics of chitosan; it results in increased drug internalization in target cells [59,60,61]. A significant barrier to its implementation is that it is soluble only in acidic media. The broad amino and hydroxyl groups serve as target groups for chemical changes to increase solubility. CS has good biocompatibility, low immunogenicity and biodegradability. Under in vivo enzymes, CS will decompose into water and carbon dioxide and become an endogenous species, ensuring no harmful effects of degradation products. Due to its increased solubility at a slightly acidic pH such as that present in the tumour microenvironment, CS is commonly used to develop pH-sensitive DDS [62,63,64,65].

Biopolymeric NPs such as chitosan (CS) are mainly used for cancer therapy because of their benefits as drug delivery vectors:

1. Cost-effectiveness [66].
2. The high transport ability of CS for hydrophobic and hydrophilic medicines, which enhances medication delivery and therapeutic effectiveness [67,68].
3. Drug protection through encapsulation in the core of NPs [69].
4. The ability to boost intracellular accumulation (the drug bioavailability in the intracellular environment) [67,70].
5. Controlled release properties [68,71].
6. Enhancement of the therapeutic efficacy of therapy, especially in tumor therapy, through passive targeting or enhanced permeation and retention (EPR) effects [70].
7. Mechanical properties, targeting ability, and mode of drug release that can also be controlled by modifying the structure of natural materials with polyamines, small molecules, and targeting ligands [56].
8. Inhibitory effects on tumor cell proliferation, tumor-associated angiogenesis, and metastasis, thus exhibiting good anticancer activity [39,72].
9. The polymer may be made water-soluble depending on its usage [73].
10. CS may trigger innate immune responses for anticancer effects [74,75].
11. A high degradation rate which ensures the material’s safety and its protection of the environment (eco-friendly properties) [76].
12. Polymers may also be hydrophobic and adhesive; high hydrophobicity prevents blood clotting during circulation [77,78].

**Figure 3:**
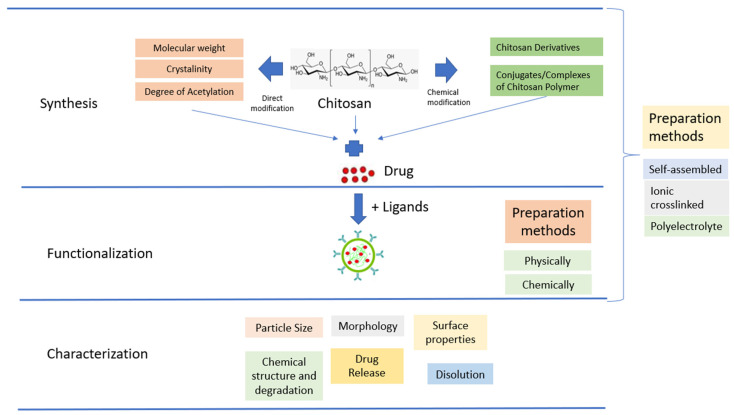
Schematic illustration of CSNPs.

The use of native CS for in vivo applications is limited by its low effectiveness, which is attributed to its instability and insufficient cellular release. To overcome these limitations, there is a need for the development of effective delivery systems that involve chemical modifications (Figure 3). Chemical modifications can be made to NPs based on CS derivatives, which possess hydroxyl, acetamido, and amine functional groups. Additionally, CS polymer conjugates/complexes and the addition of functional polymers can help mask the inherent weaknesses of CS and enhance its transfection efficiency. Modified CS can be used to adjust the carrier’s functionality and delivery properties to suit the specific application requirements. The primary goal of modifying CS is to enhance its solubility, which may lead to a wider range of potential applications [68].

CSNPs can be categorized into three groups depending on how they are prepared. The first group is self-assembled, wherein hydrophobic chains gather spontaneously to create reservoirs for drugs that dissolve in water. Meanwhile, hydrophilic chains form a shell around the nucleus that is affected by the aqueous phase [79]. The second group is ionic crosslinked, wherein ionic crosslinked NPs are created using electrostatic interaction between the cationic property of CS and a polyanionic crosslinker such as TPP, CaCl_2_, or Na_2_SO_4_. The physical characteristics of these NPs can be adjusted by varying the ionic crosslinking processing parameters; this affects the encapsulation efficiency and drug release [80]. The third group is polyelectrolyte complexes; these involve mixing CS with a negatively charged polyelectrolyte to form a CSNPs polyelectrolyte complex. This procedure is simple, and does not require toxic reagents. When mixed in solution, the polymer chains interact to create a strong but reversible electrostatic network without using crosslinking [68].

In the process of measuring CSNPs, sample preparation is a crucial step that requires considerable effort to minimize agglomeration which can negatively impact the measurement result of constituent nanoparticles [81]. The important characteristics of CSNPs are particle size, shape, morphology, surface properties, dissolution, stability, and degradation. Particle size is measured using either non-imaging or imaging-based techniques. Non-imaging techniques are rapid and repeatable, but do not provide shape information; meanwhile, imaging-based techniques such as SEM, TEM, and AFM are time-consuming but provide shape and morphological information. Particle shape determination requires imaging techniques such as SEM, TEM, and AFM [82]. Drug release from NP dosage forms can be assessed using various methods such as sample and separate, continuous flow, and the dialysis method. The DM is the most commonly used method for determining drug release from NP dosage forms. Dissolution is another important aspect, as higher dissolution rates can improve drug absorption, and it is also critical for the transformation and toxicity of NPs in aquatic environments [83]. Stability and degradation can be assessed by measuring the activation energy (Ea) using the kinetic, thermal degradation method, and the Ea value can indicate the product’s stability and extended shelf-life over a range of temperature conditions [32].

### 3.2. Mechanism of CSNPs for Drug Delivering

Chitosan nanoparticles are formed by the interaction of positively charged chitosan with negatively charged molecules such as DNA, RNA, and drugs, via electrostatic interactions [1,59,84,85,86,87]. These nanoparticles can be administered to the body via various routes such as oral, topical, or injection [78,88,89]. Upon administration, chitosan nanoparticles protect the encapsulated drugs from degradation and enhance their absorption and distribution [75,77].

To target specific tissues or cells, chitosan nanoparticles can be functionalized with ligands or antibodies that recognize and bind to specific receptors on the target cells [56,90]. This strategy improves drug delivery to the target site and reduces off-target effects.

Chitosan nanoparticles can also be designed to achieve controlled drug release by using different encapsulation strategies. For example, drugs can be encapsulated within the chitosan matrix, loaded onto the surface of the nanoparticles, or attached to the surface of the nanoparticles via chemical linkers. The release rate of the drugs can be controlled by varying the size and charge of the nanoparticles or modifying the chitosan matrix with different chemical groups [68,91,92].

### 3.3. Modification Strategies of CSNPs for BC Therapy 

Natural chemotherapeutic medications are more successful and less hazardous in cancer therapies, according to chemical and pharmacological studies. Thus, natural chemotherapeutic medication development is a prominent pharmaceutical issue [93]. The big challenges in the development of anticancer drugs are:

1. Anticancer drugs are generally hydrophobic. Small-molecule hydrophobic drugs can be quickly removed from the tumor site [94,95].
2. Poor solubility makes it difficult to dissolve and release the drug from the tumor [94,95].
3. They have a lower half-life and subtherapeutic tumor concentrations. SiRNA, microRNA, and oligonucleotides for cancer treatment degrade systemically, lowering t ½ [96].
4. Oral administration is preferable, easy, and cost-effective. This route must pass across multiple biological barriers, such as the blood-brain barrier and tight junction barrier, and be quickly destroyed by digestive fluids and the liver [97].

Given the enormous cost of drug development, exacerbated by high failure rates, anticancer drugs are usually expensive. The search for a new delivery system is expected to find a solution that can increase the effectiveness and efficiency of the use of existing cancer drugs [98,99]. In building this delivery system, there are several strategies offered by chitosan, from various studies that have been conducted:

1. CSNPs may encapsulate or conjugate chemotherapeutic medicines, therapeutic gene nucleic acids, photosensitizers, and cytokines for more reliable cancer target treatment [1].
2. Cancer tissue can be targeted either passively or actively [100,101].
3. Designing CSNPs according to delivery system factors, such as size and size distribution, drug loading capacity, and stability, is possible [102].
4. Chitosan has three groups: amino, acetamido, and hydroxy groups; they can provide derivatives of increased solubility and outstanding anticancer activity, offering bioavailability in cancer cells by utilizing sustained release. They also offer increased permeation, transfection, and gelation in situ [103], and easy in vivo biodegradation [104].
5. Multi-functional CSNPs can continue to offer many new opportunities for biomedical applications because they have the ability to interact with complex cellular functions in new ways [50]. There exists a multifunctional DD that combines high specificity against cancer cells with endosomal escape ability.
6. Surface modification of CSNPs can be carried out on polymers through physical or chemical methods. Surface modification of CSNPs enhances their tumor-targeting ability through different mechanisms such as a receptor or carrier-mediated transcytosis [105]. CSNPs were more effective than PLGA NPs because they targeted MCF-7 cells [102].
7. Modification of chitosan can use variations in molecular weight and the level of acetylation, which will provide different properties according to the needs of the drug delivery system [104].
8. Chitosan can be used as a solubility-enhancing polymer backbone. Advances in polymer chemistry have led to the creation of smart polymer systems [105].
9. Polymers used for drug administration can respond to stimuli such as temperature, light, or pH. Stimulus-responsive polymers may modify cell adhesion to boost gene expression or enzyme activity [96].

Chitosan-based gene delivery systems have attracted considerable attention as a promising alternatives to viral-based gene therapy due to their biocompatibility, low toxicity, and ease of functionalization [106,107,108]. Scientists have been exploring different ways to deliver genetic material into cells for gene therapy, which are categorized into two groups: viral and non-viral methods [109]. Viral vectors have been used in the majority of gene therapy clinical trials due to their high transfection efficiency and gene expression levels. However, they also have significant drawbacks such as immune responses, toxicity, immunogenicity, low loading capacity, and inflammation. Non-viral methods, on the other hand, are gaining increased attention because they can avoid many of these drawbacks. One promising non-viral method is the use cationic polymers such as chitosan, which have strong gene complexation and high transfection efficiency, for gene delivery. However, chitosan-based gene delivery carriers still face challenges such as poor water solubility, charge reduction at physiological pH, and poor targeting capability, which hinder their clinical translation [106,110,111,112]. Low-molecular-weight chitosan and a low N/P ratio (the ratio of the amine groups of chitosan to the phosphate groups of DNA) are more suitable for designing chitosan-based nonviral vectors for gene therapy. This is because the physicochemical and biological properties, as well as the stability of nanoparticles formulated with low-molecular-weight chitosan, are better than those formulated with higher-molecular-weight chitosan. [112].

### 3.4. Stimuli-Responsive NPs

A “smart” DDS may initiate the release of drugs near tumors or malignant cells. Systems may be regulated both internally (through the pH of the tumor microenvironment, glutathione (GSH) redox potential, and specific overexpressed enzymes) and externally (through magnetic, ultrasonic, thermal, microwave, electrical, and photochemical signals) [49,105]. Exploiting physiological variations between tumor and healthy tissue facilitates the development of stimuli-responsive DDS [38]. This reduces the systemic toxicity of chemotherapeutic drugs and restricts healthy tissue exposure to cytotoxic drugs. Quantitative mechanical description of active components plays a crucial role in their development and application, allowing the design of complex devices and the engineering of the microstructures of materials according to their intended functionality [113].

Nonetheless, dual or multi-responsive polymers have garnered an increasing amount of interest, since several applications for these intelligent materials require reactions to multiple external stimuli [113,114].

Light can manipulate polymers without human contact or mechanical methods. Scientists can modify the molecule’s shape and dipole moment. This affects wettability, permeability, and color. These may help or trigger drug release around the tumor. To replicate complicated biological systems, one must understand stimuli responsiveness to recognize particular changes and react predictably. Polymer reactions might occur concurrently or sequentially from preparation through transport routes to cellular compartments [115].

### 3.5. Multifunctional Delivery Systems 

The chemical synthesis of nanomaterials has been a powerful force behind advances in nanoscience and nanotechnology [116]. Multifunctional delivery systems include diagnostic imaging, targeted medicine delivery, and controlled drug release. These particles allow active cancer monitoring throughout chemotherapy, which improves treatment control. Multifunctional delivery systems can provide and monitor combined drug therapy to improve cancer treatment [35] and measure pharmacokinetics and biodistribution in real time [51].

## 4. CSNPs and Mechanism Anticancer Action

CS has several active functional groups, offering more diversity and modification opportunities. NPs operate as nanocarriers in cancer therapy, containing anticancer drugs. The potential of nano-therapeutics relies on the proper unloading of drug cargo to the target region, which is mediated by six pathways: targeting, cellular uptake, drug release, MDR, cytotoxicity, and cell death. Methods such as emulsification, solvent evaporation/extraction, nanoprecipitation (solvent-displacement), salting-out and the supercritical anti-solvent method have been used for the preparation of PNPs. 

Chitosan and its derivatives can inhibit the growth of various cancer cells induce apoptosis through increasing the concentration of calcium ions, the level of ROS and the mitochondrial membrane potential [1], and inhibit cancer cell migration and invasion. Chitosan can also modulate various signaling pathways involved in cancer progression, including the PI3K/Akt/mTOR pathway [117], the MAPK/ERK pathway [118]., and the NF-κB pathway. Chitosan and its derivatives can induce cell cycle arrest and apoptosis in cancer cells by regulating these pathways. Chitosan oligosaccharide may improve the efficacy of existing chemotherapies by inhibiting programmed cell death ligand 1 (PD-L1) expression and promoting T cell-mediated immune killing in tumors [1].

### 4.1. Targeting 

The role of CSNPs in targeting systems has been discussed in previous reviews [56]. They can be involved in both passive (based on the enhanced permeability and retention effect of cancer targeting) and active (receptor-mediated or stimuli-responsive cancer targeting) drug delivery systems for potential cancer therapy [119]. Passive and active targeting techniques for systemic medication administration target solid tumors without harming healthy cells/tissues [4]. 

EPR-based passive targeting may improve nano-treatment tumor aggregation and persistence by leaking tumor arteries which generate the EPR effect, thereby enabling the NPs to enter and reside in the tumor core. The holes in the leaking tumor vasculature range in size from 380 to 780 nm. NPs below this threshold may target cancer cells. Small particles less than 80 nm may readily permeate the tumor by passive diffusion and are pushed back into the circulation by the high interstitial fluid pressure inside the tumor. NPs must first pass through the tumor vasculature (usually a leaky microvasculature) and then extravasate into the tumor interstitial space, where tumor heterogeneity and hypoxia might limit real effect [4]. 

NPs often combine passive and active targeting; they concentrate more on cancer tissue than on normal cells and tissues, reducing side effects and therapeutic doses [35,105,120].

Multiple types of ligands are conjugated to the particle’s surface as part of the multiple-ligand targeting strategy. Due to the coupling of two stimulus-based systems in one matrix, it is believed that multiple stimulus-responsive materials should be more efficient than single stimulus-responsive materials [121].

This explains TNBC’s dismal 5-year survival rate. CD44+/CD24 BCSCs are responsible for TNBC resistance and relapse. Innovative therapeutics targeting TNBC stem cells and vulnerable cells are required. BCSCs are epithelial- or mesenchymal-like. HA binds BCSCs’ CD44 receptors [96].

### 4.2. Cellular Uptake 

The efficiency of cellular absorption is essential for the effectiveness of nanotherapy [122,123]. The cellular absorption of NPs is determined by some variables, including particle size, charge, hydrophilicity, and the presence of ligands. CSNP provides advantages to these factors; the size of CSNP and its derivatives can reach 100 nm or less. The existence of a positive charge from CSNPs and their derivatives causes interactions with negatively charged cell membranes [77,124]. Zeta potential plays an important role. Positively charged NPs prefer to be internalized quickly and intensely by negatively charged cancer cells due to electrostatic interactions. To further enhance penetration into cells, ligands can be attached to CSNPs. NPs are predicted to be absorbed by cancer cells before releasing their therapeutic payload. 

Increasing drug levels in malignant cells reduces negative effects. Endocytosis is energy-dependent and actin-mediated [125]. In the clathrin-mediated route, 60 to 200 nm particles enter the cell through vesicles formed when clathrin-coated pits invaginate [126]. Caveolae carry particles above 200 nm. They interact with G-protein-coupled receptors, receptor tyrosine kinases, and steroid hormone receptors in caveolae pathways, causing membrane invaginations. NPs with lipid-binding characteristics may diffuse into cells. Non-specific binding in systemic circulation may affect NPs’ surface characteristics, resulting in different in vivo and in vitro behavior.

### 4.3. Drug Release 

The starting point for the course of the therapeutic action of an NP-based drug delivery system is drug release. Drug release from CSNPs, unmodified chitosan polymers, and chitosan with various modifications has been discussed in a previous review [68]. CSNPs can controllably transfer active substances, enhancing therapeutic efficacy. CSNPs have a small size and substantial surface area for targeted delivery. CS’s hydrogen bonding and cationic charge in acidic conditions make it a good DDSs candidate.

The release of medicines from CSNPs followed a typical bi-phasic pattern, with a rapid initial release followed by a delayed, more pH-dependent release [68]. Microenvironmental acidification and other metabolic crosstalk are interesting possibilities. Increased glucose metabolism lowers the pH in the microenvironment due to lactate secretion. The acid-mediated invasion hypothesis suggests that H+ ions secreted from cancer cells diffuse into the surrounding environment and alter the tumor–stromal interface, allowing enhanced invasion [127]. The pH is lowered to 6.0–7.0 as a result of glycolysis, which creates a slightly acidic environment in a low-oxygen environment. Additionally, the pH of lysosomes within cancer cells is significantly lower, only reaching 5 [128,129]. The Warburg effect can provide advantages for cell growth in a multicellular environment. Chitosan-CD44 interactions promoted the absorption of chitosan-coated nanosystems. [130]. 

### 4.4. MDR

**Figure 4:**
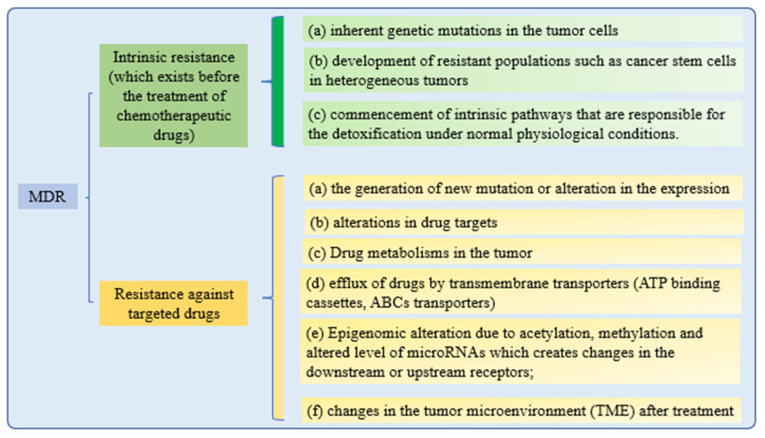
Mechanisms of drug resistance.

Cancer cells’ drug resistance causes the failure of anticancer therapy. Increasing drug efflux, evading drug-induced apoptosis, and activating DNA repair pathways can drive chemotherapy resistance [131]. Multiple attempts to overcome MDR in cancer have failed in recent years. Understanding MDR and cellular reprogramming may help us to overcome cancer drug resistance and improve cancer treatment (Figure 4). Cancer multidrug resistance may be addressed using novel anticancer drug candidates, molecular targets, and revolutionary DDS [9].

All of these drug resistance mechanisms may cause multidrug resistance in cancer. While retaining the effectiveness of the treatments, nanotechnological strategies are being developed to reduce the toxicity of targeted therapies to healthy tissues. These methods are based on a nanoparticle delivery system (nanocarriers) that enhances drug–site contact time and elimination time, while decreasing drug resistance [132].

From Table 1, it can be seen that CSNPs can reduce MDR through various mechanisms. CSNPs can increase the effectiveness of anticancer drugs in the following ways:

1. NPs can release cargo before reaching target cells. Therefore, they need targeted ligands that can recognize and bind tumor cells without harming healthy ones. Active targeting facilitates drug endocytosis, circumventing cytotoxic drug efflux ABC transporters [3].
2. Nanomedicines can encapsulate many molecules, be targeted, and encourage controlled release, thereby boosting combination therapy. The extracellular matrix (ECM), cytokines, and stromal cells impact tumor cell invasion through the stroma. In several cancers, fibrotic stroma inhibit medication distribution and penetration. OA can inhibit matrix metalloproteinases (MMPs), which may reduce tumor stromal cell fibrosis. All these studies suggest OA could boost cancer chemotherapy [60]. The encapsulated flavonoid silybin can operate as an MDR inhibitor by inhibiting the P-gp pump’s “drug-pumping” action [130].
3. CSNPs bypass MDR because they enter cells by “stealth endocytosis,” preventing drug molecules from being identified by P-glycoprotein (P-gp). Perinuclear NPs can release drugs to avoid efflux pumps [133].

**Table 1:** Nanocarriers for overcoming multidrug resistance in BC.

Drug	Polymer CS or Derivated	Cancer Model	Targeting Ligand	Mechanism	Results	Ref.
Doxorubicin (DOX)	Thiolated glycol-CS (tGCS)	Adriamycin-resistant MCF-7	siRNA P-gp	Functional siRNA release, in-vivo P-gp downregulation	Subtherapeutic DOX dose inhibited tumor development	[134]
DOX and oleanolic acid (OA) co-delivery	Folic acid-CS	MDA-MB-231	Folate	OA can inhibit MMPs. MRP and P-gp inhibition reverse MRP-mediated efflux	High uptake, and longer circulation than free DOX, reduced DOX-induced tissue damage	[135]
DOX	Folic acid-hydroxypropyl-chitosan (HPCS) and oligodeoxynucleotides (ODN)	KB-A-1 DOX-resistant cells	Folate	Inhibition of the MDR 1 gene levels and P-gp levels in vitro and in vivo	Comparatively, ODNs inhibited tumor development by 35%	[136]
DOX, Paclitaxel (PTX), and Silybin.	PLGA NPs, followed by a double layer of lipids and chitosan.	MDA-MB-231 A-549	CD44s	Chitosan and CD44 interactions were the main way that CSNPs were taken up. Silybin can block the P-gp pump to act as an MDR inhibitor	NPs cut the size of the tumor by five times compared to the control group without causing obvious cell death	[130]
Doxorubicin hydrochloride and Tariquidar (TQR)	Biotinylated carboxymethyl chitosan hybrid	MCF-7/ADR cells	Biotin as a targeting ligand	TQR prevents P-gp-mediated drug efflux and boosts intracellular drugs. Tumor cells absorbed more biotin ligands	Better cell uptake and nuclear localization than free DOX	[133]
Ligustrazine (LZ)	Folate-chitosan NPs (FA-CS-NPs)	MCF-7 (folate receptor-positive) and A549 (folate receptor-negative) cells	Folate	Ligustrazine (LZ) improves the sensitivity of multidrug-resistant cancer cells to chemotherapeutic agents	High cellular uptake specificity by FR-expressing cells. FA-CS-LZ-NPs, are a promising candidate for overcoming MDR	[137]
Curcumin (Cur) and DOX	CS-based NPs using genipin (crosslinker)	MCF-7/ADR		Intact positively charged NPs (or an amino group) to the negatively charged DNA in the nucleus	Extended circulation time, enhanced tumor targeting effectiveness, increased tumor inhibition efficacy and decreased expression of MDRP	[138]
siRNA and DOX	CS-coated PF127-TPGS mixed micelle based	4T1 resistant 4T1	Folate	Improved anticancer effectiveness and blood circulation	Increased cytotoxicity in native 4T1 and multidrug-resistant 4T1 vs. free DOX	[139]

### 4.5. Cytotoxicity

**Figure 5:**
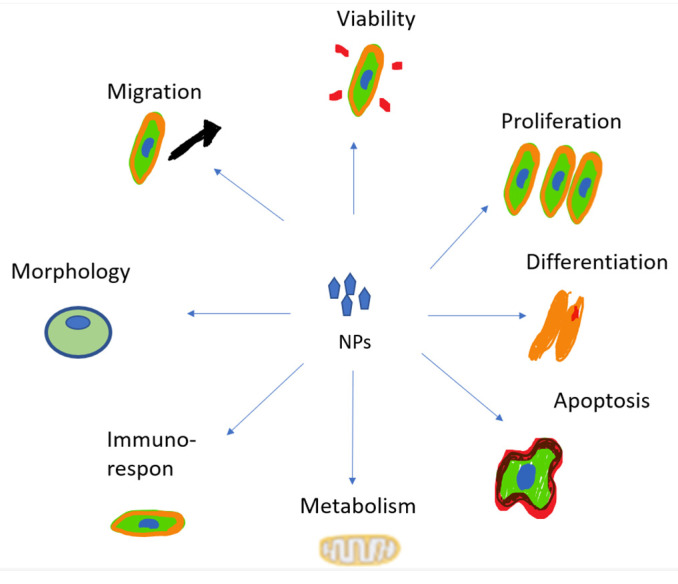
The toxic effects of NPs.

The increase in cytotoxicity is the result of the previous processes of cellular uptake, drug release, and reduction of MDR. Both can be achieved by targeting patterns from CSNPs. The toxic effects on cells induced by NPs’ uptake are mainly due to the reasons shown in Figure 5 [140]. 

**Figure 6:**
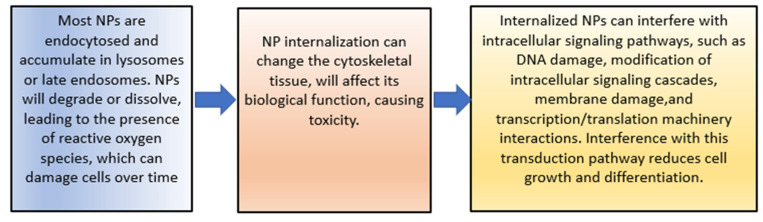
The toxic effects of NPs.

The toxicity of NPs can also be caused by damage to cell membranes. The process is described in Figure 6.

However, the greater surface area to volume ratio of these particles causes their higher chemical reactivity and results in increased production of reactive oxygen species (ROS). Indeed, the NPs surface area is a key factor in their intrinsic toxicity because of the interaction of their surfaces with biological systems [141]. Non-specificity and systemic toxicity are common causes of chemotherapy’s undesirable effects on healthy cells and limited therapeutic impact and morbidity. Cancer cells’ resistance to apoptosis and cytotoxic drugs causes 90% of therapy failure. Thus, a new discipline in BC treatment is needed to increase the cytotoxic effectiveness of conventional anticancer medications [37]. Nanocarriers with particle size less than 150 nm (or 200 nm) are thought to be particularly useful and successful for cancer therapy because they passively target tumor cells via enhanced permeability and retention. A ZP larger than 30 mV has the potential to stabilize colloidal preparations, preventing particle aggregation [142]. The advantages of CSNPs can be seen in Table 2; various compounds can be delivered, and their cytotoxicity can be increased. By providing ligands as active target agents, they will reduce the side effects of anticancer drugs.

### 4.6. Cell Death

There are two main apoptotic mechanisms: the death receptor and mitochondrial pathway [149]. The intrinsic mitochondria-mediated route is usually disrupted in cancer and can be triggered by lysosomal/ER stress, metabolic stress, DNA damage, ionizing radiation-induced cellular stress, hypoxia, heat, cytokine deficiencies, and chemotherapeutic drugs. The mitochondrial apoptotic pathway is the most frequently inhibited apoptotic pathway in cancer cells. Cyt c release activates the caspase activation cascade in the mitochondrial apoptotic pathway. There exists a Cyt c drug delivery device that targets the mitochondrial apoptotic pathway to promote cancer apoptosis. [40]; inducing intrinsic or extrinsic apoptosis is a promising cancer therapeutic method.

Table 3 shows the ability of CS NPs to deliver various drug compounds and to increase their effectiveness. Positive NPs may cross the mitochondrial negative potential barrier, and lower mitochondrial membrane potential (MMP) by causing mitochondrial malfunction or death [84].

Non-apoptotic cell death involves halting the cell cycle and antiangiogenic mechanisms, thus producing endoplasmic reticulum stress pathways and causing cell cycle arrest through p53-mediated signaling and the Tp53/p21 pathway. The endoplasmic reticulum may also affect tumor cell apoptosis, autophagy, and treatment resistance. The endoplasmic reticulum is a target for metallodrugs. Ruthenium and iridium complexes activate caspase proteins, which accelerate tumor cell death.

Autophagy-related (ATG) proteins limit cancer cell death by regulating autophagy. Necrosis and apoptosis are different processes. Autophagy is an internal catabolic mechanism in which autophagosomes engulf and destroy cellular components. Autophagy recycles cytoplasmic contents and maintains cellular homeostasis. Autophagy is a critical cell death mechanism involved in mammalian physiology, including tumor suppression and mental disorders. ROS causes apoptosis and necrosis (ROS) [150].

Programmed cell death (PCD) is induced by intracellular mechanisms. PCD is triggered to sculpt or remove structures, control cell populations, and eliminate unneeded or malfunctioning cells in tissue formation and homeostasis. Thus, aberrant PCD regulation is linked to malignancies and neurological disorders. Autophagy and apoptosis are self-regulatory systems that help cells maintain homeostasis [85]. In some situations, autophagy and apoptosis can cooperate to kill cells [150].

When entering a complex biological system, NPs will interact immediately with surrounding biomolecules. This results in the formation of a protein “corona” on the NPs’ surface. Researchers have observed that this corona might alter the biodistribution, stability, pharmacokinetics, immune response, and toxicity of polymeric nanoparticles. In addition, several in vitro and in vivo animal studies have been conducted to comprehend the effects of polymeric NPs on certain cancer therapies.

**Table 3:** Nanocarriers for enhancing cell death in BC.

Drug	Polymer CS or Derivated	Cancer Cell Model	Mechanism	Results	Ref.
DOX	CS- montmorillonite (MMT)-quantum dots	MCF-7	pH 5.4 controlled DOX release, whereas pH 7.4 had none, suggesting fewer negative effects	NPs had greater cytotoxicity than free DOX in MCF-7 cells	[151]
Mebendazole (MBZ)	Folic acid-CSNPs	4T1 murine TNBC	pH-sensitive CS-FA-MBZ NPs enhance MBZ release in the tumor microenvironment	15 days after implantation, CS-FA-MBZ implants degraded entirely, reducing tumor volume	[72]
Seleno	Seleno-short-chain CS	MCF-7 BT-20	SCC caused apoptosis in MCF-7 and BT-20 cells in vitro by upregulating Bax and downregulating Bcl-2	MCF-7 and BT-20 cells could undergo in vitro apoptosis when exposed to SSCC via the mitochondrial route	[152]
Gold NPs	Chitosan-gold NPs	MCF7 MDA-MB-231	Activation of the p53-p21-mediated cell cycle arrest is concurrent with activation of the Bax-Caspase9-Caspase3-PARP1 axis	Extremely effective against BC cells while having no obvious harmful effects on normal cells	[39]
Rutin	Rutin-CS nanoconjugates	TNBC cells	Apoptotic cell death causes DNA synthesis to stop, and DAPI fluorescence micrographic analysis	Triple-negative BC apoptosis	[39]
ZnO-NPs	CS-ZnO NPs	MCF 7	Significant cell cycle arrest at a particular stage of G2/M was achieved with the nanocomplex treatment in a dose-dependent manner. Finally, it was observed that the apoptotic genes and protein expressions of the MCF-7 cell line were up and down-regulated with the treatment of Ch-Ap-ZnONPS when compared to normal cells	The spherical and cubic nanocrystals were found to be lethal against MCF 7 cells whose IC50 value was 42 μg/mL, on MTT assay, in dose-dependent manner (20–80 μg/mL),	[39]
Doxorubicin	CS-protamine NPs	MDA-MB-231	CPNPs-DOX downregulates Bcl-2 relative to free DOX and control.	Treatment with NPs reduces cell viability/count	[153]
Ascorbic acid and Oxaliplatin	Non-PEGylated and PEGylated CS NPs (CS NPs)	MCF-7 cells	AA stimulates the internal apoptotic process, whereas OX activates the extrinsic route. Evidence shows that the two pathways are interconnected and that chemicals in one may impact molecules in the other	PEGylation improves AA and OX’s apoptotic effects on MCF-7 cells	[149]
Chitooligosaccharides (COS)	Chitooligosaccharides (COS) D3–7 (D-deacetylated unit) and A5 (A-acetylated unit)	BT-474 SUM-159	Apoptosis is promoted by the strong reduction of phosphorylation of EGFR and its downstream signaling pathways FAK, AKT, and MAPK	In a dose-dependent manner, COS significantly reduces the viability of BC cells	[154]
Curcumin	Iron (II, III) oxide (Fe_3_O_4_) NPs coated with carboxymethyl-CS	MCF-7 MDA-MB-231 human fibroblast	In MCF-7 cells, combinational therapy-induced cell death (64.51 percent) and sub-G1 cell cycle arrest were observed. In addition, MCF-7 cell proliferation may be inhibited	The IC_50_ level of MNP-CMC-CUR has been dramatically reduced when compared to free curcumin, as has the metabolic activity of the cells (p 0.05)	[155]
Curcumin and Chrysin	Alginate-CS hydrogel	T47D	G2/M causes arrest of the cell cycle	NPs drastically impair viability and trigger apoptosis in cells	[156]

## 5. Perspective

Nanotechnology has led to medical discoveries and is now an integral part of healthcare systems. CSNPs can boost the efficiency of anticancer medications by improving chemotherapeutic delivery to cancer cells, drug uptake into tumor cells and drug accumulation, inhibiting MDR proteins such as P-gp, increasing drug bioavailability, and inducing apoptosis. Combination therapy and novel medications may help overcome resistance and minimize negative effects, according to recent studies. Dual responsive nanocarrier systems show promise as chemotherapeutic drug delivery vehicles, but additional study is needed to confirm their usefulness in animal models.

Multidisciplinary methodologies and industry-academic partnerships should boost this sector’s research. Future medical improvements depend on commercializing scientific results. Nanotechnology could revolutionize personalized medicine. Combination approaches may alleviate chemotherapy’s limitations and improve treatment outcomes.

Development of imaging-targeted distribution and controlled-release medication delivery devices is underway. Concurrent targeting of ligands to several overexpressed receptors on malignant cells improves particle selectivity for malignant cells while restricting the absorption of non-malignant cells. Increased collaboration between oncologists and engineers may lead to more therapeutic NPs with the optimum drug dose and release qualities. Data science and innovative process engineering can produce unique medications. Because of their ability to target specific stimuli and multitask, they are a popular research topic. Surface characteristics of particles can be adjusted by pegylation to promote biocompatibility and decrease cytotoxicity.

CSNPs can improve biocompatibility, circulation time, targeted delivery, drug release kinetics, and drug degradation. New strategies for drug–polymer conjugation and nanomaterials engineering will widen the nanoparticle platform for designing better treatment regimens. Despite its benefits, the slow degradation rate of some CSNPs with chemical modification limits their clinical utility. Nanomaterials that interact with tissues/organs raise safety concerns. Understanding NPs’ biological clearance time is crucial [96].

## 6. Conclusions

In this study, we highlight drug conjugates in CSNPs with augmented multifunctionality that are decorated with stimuli and targeting sites shown to enhance their higher anticancer efficacy compared to free drug compounds. Diverse polymeric co-delivery systems have been obtained that exhibit high anticancer efficacy, especially in multidrug-resistant cancers. Targeting ligands can be attached to particles to promote active targeting and drug endocytosis of targeted cancer cells. The controlled release of anticancer drugs from particle carriers can be achieved using pH, enzymes, or thermoresponsive particles or conjugates. However, many challenges remain associated with co-delivery approaches, including loading, capacity, stability, release kinetics, biocompatibility, and tumor-targeting efficacy.

## Figures and Tables

**Table 2:** Nanocarriers for enhancing cytotoxicity in BC.

Drug	Polymer CS or Derivated	Cancer Model	Mechanism	Results	Ref.
α-mangostin (AMG)	Depolymerized chitosan	MCF-7	Enhance the physicochemical characteristics	Enhancement of the cytotoxicity of AMG	[92]
DNA delivery	An iron oxide core coated with low-molecular-weight (800 Da) polyethyleneimine crosslinked with chitosan	SKBR3, MCF7 4T1	Nano-size, positive surface charges for DNA condensation, protection, and serum stability	The ability to deliver DNA for DNA transfection in vitro	[143]
Z. multiflora EO	Low -molecular-weight CS	MCF-7 MDA-MB-468	Increases cellular uptake, solubility, and biological and pharmacological activities	CSNPs containing Z. multiflora EO were more powerful than non-formulated Z. multiflora EO in prior investigations	[143]
Tamoxifen (TMX)	Hyaluronic acid-coated chitosan NPs	MCF-7 TMX-resistant MCF7	HA-conjugating to CD44 receptors increased nanoparticle drug uptake	NPs with acidic pH (5–6) released more TMX at pH 7.4. HA-CS NPs were more cytotoxic than CS NPs and free drugs	[102]
Turmeric oil (TO)	CS-alginate NPs (CS/Alg-NPs)	MDA-MB-231 MCF-7	NPs-induced apoptosis in normal and malignant cells can proceed via ROS production, activating caspase 9, and causing the mitochondrial intrinsic apoptosis pathway	Increasing BC cell cytotoxicity	[144]
Human growth hormone (hGH)	Gum Arabica chitosan NPs	normal (HUVEC) MDA-MB-453	Increase doxorubicin-loaded CSNP toxicity by binding to BC target proteins	Dual-loaded CSNPs had a stronger anti-proliferative effect against MCF-7 than doxorubicin-loaded CSNPs	[142]
Cisplatin	Cisplatin-loaded CSNPs and cisplatin-loaded CSNPs surface linked to rituximab	MCF-7	The inhibition was ascribed to simple passive penetration via cell membrane pores and delayed breakdown inside cells, resulting in sustained action at the lowest drug dose	A novel cisplatin–DNA tetrahedron-body-expressed nano drug exhibited more cytotoxicity than cisplatin against HER2-overexpressing BC cells	[145]
Peganum harmala smoke extract (PSE)	PLGA-NPs coated with folic acid-CS (PCF-NPs)	MCF-7	MCF-7 cells undergo apoptosis when P53, Cas-3, and Cas-9 genes are upregulated	Selective toxicity on MCF-7 cells	[146]
Stattic (S)	CS-coated-poly(lactic-co-glycolic acid)	MDA-MB-231 4T1 cells	Increased accumulation in the mouse primary tumor and newly formed metastatic foci with high angiogenesis activity	Post-entrapment, the drug’s antimetastatic characteristics improved physicochemically, in vitro and in vivo	[147]
Co-delivery of MTX and STAT3 siRNA	Mesoporous silica NPs were functionalized with CS	MCF7 cells	Additional free MTX boosted cellular absorption of modified NPs, involving the DHFR receptor. NPs with and without drugs have variable protein corona compositions, influencing cellular absorption	BC cell viability was dramatically reduced as compared to single treatments alone	[148]
